# Measurement of Key Constructs in a Holistic Framework for Assessing Self-Management Effectiveness of Pediatric Asthma

**DOI:** 10.3390/ijerph16173060

**Published:** 2019-08-23

**Authors:** Pavani Rangachari, Kathleen R. May, Lara M. Stepleman, Martha S. Tingen, Stephen Looney, Yan Liang, Nicole Rockich-Winston, R. Karl Rethemeyer

**Affiliations:** 1 Department of Interdisciplinary Health Sciences, College of Allied Health Sciences, Augusta University, Augusta, GA 30912, USA; 2 Division of Allergy-Immunology and Pediatric Rheumatology, Department of Pediatrics, Medical College of Georgia, Augusta University, Augusta, GA 30912, USA; 3 Department of Psychiatry & Health Behavior, Medical College of Georgia, Augusta University, Augusta, GA 30912, USA; 4 Georgia Prevention Institute, Medical College of Georgia, Augusta University, Augusta, GA 30912, USA; 5 Department of Population Health Sciences, Medical College of Georgia, Augusta University, Augusta, GA 30912, USA; 6 Department of Pharmacology & Toxicology, Medical College of Georgia, Augusta University, Augusta, GA 30912, USA; 7 Rockefeller College of Public Affairs & Policy, University at Albany, State University of New York, Albany, NY 12222, USA

**Keywords:** asthma management, pediatric asthma, evidence-based guidelines, self-management effectiveness measures, medication adherence, holistic framework, practice improvement

## Abstract

The 2007 U.S. National Institutes of Health EPR-3 guidelines emphasize the importance creating a provider-patient partnership to enable patients/families to monitor and take control of their asthma, so that treatment can be adjusted as needed. However, major shortfalls continue to be reported in provider adherence to EPR-3 guidelines. For providers to be more engaged in asthma management, they need a comprehensive set of resources for measuring self-management effectiveness of asthma, which currently do not exist. In a previously published article in the *Journal of Asthma and Allergy*, the authors conducted a literature review, to develop a holistic framework for understanding self-management effectiveness of pediatric asthma. The essence of this framework, is that broad socioecological factors can influence self-agency (patient/family activation), to impact self-management effectiveness, in children with asthma. A component of socio-ecological factors of special relevance to providers, would be the quality of provider-patient/family communication on asthma management. Therefore, the framework encompasses three key constructs: (1) Provider-patient/family communication; (2) Patient/family activation; and (3) Self-management effectiveness. This paper conducts an integrative review of the literature, to identify existing, validated measures of the three key constructs, with a view to operationalizing the framework, and discussing its implications for asthma research and practice.

## 1. Introduction

Asthma affects over 43 million Americans, and is associated with enormous healthcare expenditures, including an estimated $56 billion per year in direct costs [1,2]. Studies on asthma management have found that higher levels of patient activation (knowledge, skills, and confidence) in managing one’s own asthma are associated with increased medication adherence and asthma control, even in underserved rural communities [3]. However, a recent systematic review of research on supported self-management of asthma found that interventions explicitly addressing patient, physician, and organizational factors demonstrated the most improvement in clinical outcomes, compared to targeting patients or physicians, separately [4]. The review included studies from primary, secondary, community and managed care settings serving a total estimated asthma population of 800,000 people in six countries. In these studies, targeting physicians improved process (e.g., adherence), but had no significant effect on outcomes (e.g., healthcare use). Targeting patients improved some process measures, but had an inconsistent impact on outcomes. However, interventions that explicitly addressed patient, physician, and organizational factors showed the most consistent improvement in both process and clinical outcomes. These findings underscore the importance of physician (provider) and organizational (hospital and/or clinic) engagement in promoting self-management among asthma patients/families.

### 1.1. Problem of Interest

The U.S. National Institutes of Health (NIH) Expert Panel Report-3 (EPR-3) published in 2007, includes a set of evidence-based practice guidelines for asthma management [5]. EPR-3 guidelines emphasize the importance of creating a provider-patient partnership to enable patients/families to regularly monitor asthma symptoms and take control of their asthma, so that treatment can be adjusted as needed. A cornerstone of EPR-3, is the use of an “Asthma-Action Plan (AAP)” by patients and their providers. The AAP is typically a personalized written plan that explains what medicines to take and when to take them, what environmental triggers to avoid, and how to handle signs and symptoms of worsening asthma. According to EPR-3, an AAP should be developed jointly by the provider and patient, and represent an active partnership, emphasizing education and reinforcement, to enable effective self-management of asthma. Since 2009, hospitals across the U.S. have been required to provide all asthma patients with an AAP, regardless of the care setting [6]. However, the U.S. Centers for Disease Control and Prevention (CDC) continues to report major shortfalls, not only in provision of AAPs, but also in adherence to EPR-3 guidelines in clinical practice [1,7,8,9]. In summary, there is an urgent need for greater provider and organizational (hospital/clinic) engagement in asthma management.

For providers and hospitals/clinics to be more engaged in promoting asthma management, these stakeholders need a comprehensive set of resources and tools for measuring self-management effectiveness of asthma patients/families which currently do not exist. In a previous article published in the *Journal of Asthma and Allergy*, the primary author conducted a comprehensive review of the asthma management literature to develop a holistic framework for understanding self-management effectiveness in children with asthma [10]. This paper seeks to elaborate and expand upon the framework developed in the earlier article, by identifying existing, validated measures of the key constructs, with a view to operationalizing the framework, and discussing implications for asthma management research and practice.

The ultimate purpose of this effort is to create a foundation for providers and hospitals/clinics to measure self-management effectiveness in children with asthma and improve asthma care, by identifying strategies and interventions to promote ideal self-management and optimal healthcare use in children with asthma. The next sub-section discusses key lessons learned from the earlier review article, as well as our noted key constructs and variables inherent in the holistic framework (for assessing pediatric asthma self-management effectiveness), the summation of which, provides the essential background and rationale for the current review.

### 1.2. Lessons Learned from Prior Review

The longstanding availability of national guidelines for asthma management suggests that U.S. policymakers have embraced effective self-management as a viable approach for reducing the public health burden of asthma. A review of the asthma management literature however, reveals two distinct streams of thought: (1) a traditional medical model; and (2) a self-agency model. Under the traditional medical model, emphasis is placed on patient adherence to treatment prescribed by the provider, or compliance with the treatment plan [11,12,13,14,15]. Non-adherence, in turn, is viewed as a result of not only patient behavioral problems that could be addressed, but also as a consequence of socio-ecological constraints that neither the patient/family nor provider could have any immediate control over, e.g., socio-economic barriers and/or health system constraints [16,17]. Despite the initial prevalence of the traditional medical model however, efforts have been made to move toward more collaborative models of asthma management. The essence of the collaborative model is a partnership between providers and patients, with providers offering education, support, and resources for monitoring asthma, to enhance self-management. An extension of the collaborative approach is the self-agency model of asthma management, wherein patients are empowered to play an active role in understanding how they respond to illness, and planning day-to-day routines to create a sense of order. Therefore, taking control of one’s own asthma management through behavior and lifestyle modification is the essence of the self-agency paradigm [18,19,20,21].

The above discussion in turn, helps to understand that there may be two distinct sets of factors influencing self-management effectiveness of asthma patients/families:

(1) Distal factors that are beyond the immediate control of the patient/family, and would include socio-ecological factors at various levels, e.g., individual factors (demographic & biologic characteristics, asthma severity-of-illness; health literacy); socio-economic factors (insurance coverage for asthma controller medications); community-level factors (support for self-management education in schools), etc.; and
(2) Proximal factors that are within some control of the patient/family, and would include self-agency or patient/family activation in asthma management. The latter, in turn, would include behaviors and actions taken by the patient/family toward asthma management and control (e.g., regularly refilling prescription medications or refraining from exposure to tobacco smoke, to control irritant environmental triggers).

**Figure 1:**
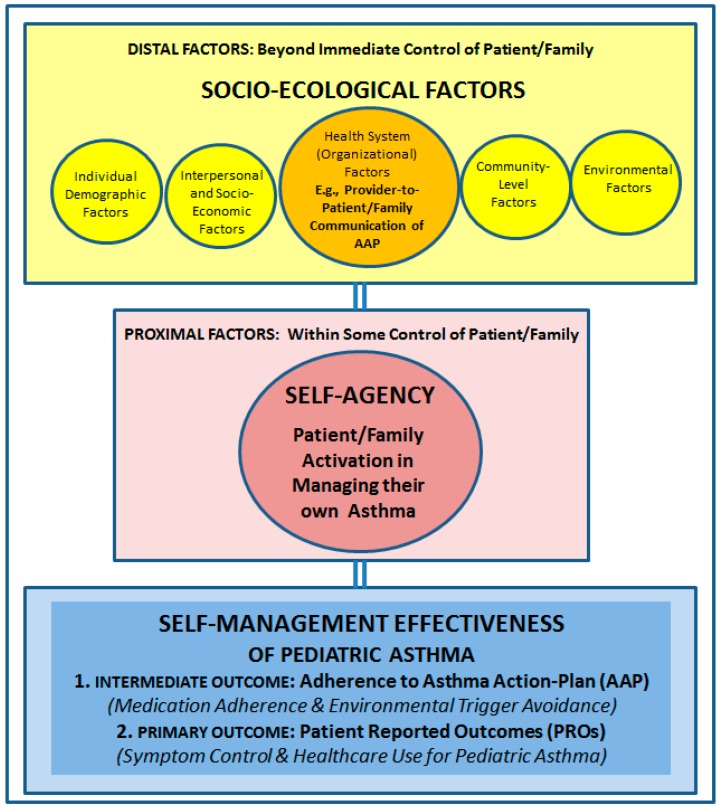
A Holistic Framework for Assessing Self-Management Effectiveness of Pediatric Asthma.

This conceptualization of self-management effectiveness in children with asthma, in turn, incorporates the key tenets of both the socio-ecological and the self-agency models of asthma management, while also accounting for a variety of patient/family demographic characteristics and risk factors [10]. Based on the above discussion, Figure 1 provides a depiction of the holistic framework for understanding and assessing self-management effectiveness in children with asthma.

As shown Figure 1, broad socio-ecological factors can influence self-agency or patient/family activation in asthma management, to consequently impact self-management effectiveness in children with asthma. Within this framework, “Health System” influences, including the quality of provider-patient/family communication of the AAP, are a component of socio-ecologic factors that are of particular relevance to healthcare providers. Explicating the role of provider-patient communication in asthma management is crucial, because it is directly actionable by providers, and therefore, may have much potential to increase provider engagement in asthma management.

### 1.3. Need for the Current Review

To summarize the above discussion, Figure 1 incorporates three key constructs for measurement: (1) Provider-patient/family communication of the AAP; (2) Patient/family activation for asthma management; and (3) Self-management effectiveness of pediatric asthma patients/families; with the latter being defined in terms of both: (a) intermediate outcomes, i.e., patient/family adherence to the AAP and (b) primary outcomes, i.e., childhood asthma symptom control and healthcare utilization. The remaining socio-ecological influences, in turn, could serve as moderating or mediating variables in the assessment of relationships among the three key constructs.

This paper seeks to elaborate and expand upon the holistic framework summarized in Figure 1, by identifying existing, validated measures of the key constructs and variables in the framework, with a view to operationalizing the framework. The key rationale for operationalizing this framework, would be to provide a concrete foundation for future research seeking to investigate relationships among the key constructs in the framework. The next two paragraphs serve to illustrate the potential value of operationalizing the framework for research, by discussing examples of hypothesized relationships among key constructs in the framework that could be investigated in future studies, to ultimately benefit asthma management practice and research. In the below discussion, H1 (Hypothesis 1), H2 (Hypotheses 2), and H3 (Hypothesis 3) refer to examples of hypotheses emanating from the holistic framework that could be investigated in future empirical research studies.

Although there is no standard definition of effective provider-patient communication, Street et al. point out that high-quality physician-patient communication is patient-centered, in that, it serves to foster information sharing, provide emotional support, manage uncertainty, create relationships, facilitate decision making, and enable self-management [22]. Some studies have found a positive relationship between physician-patient communication and health outcomes, while other studies have emphasized that the effects of effective physician-patient communication on health outcomes are mainly indirect [23,24,25,26,27,28,29,30]. Street et al. have provided a model to describe how high-quality physician-patient communication could influence health outcomes via direct and indirect pathways. They suggest that immediate outcomes linked to physician-patient communication are: patients’ understanding, trust, agreement, involvement, and motivation [22]. These immediate outcomes affect intermediate health outcomes such as self-care skills, which in turn, are known to ultimately affect health and wellness. Therefore, the literature suggests that

**H1.**  *High-quality physician-patient communication can empower patients to engage in activities needed to manage and control their asthma, thereby improving their self-care skills.*

Patient action towards managing chronic disease, in turn, has been described in the literature as “Patient Activation.” According to this literature, active patients are those who take action towards self-management (e.g., initiate discussions with provider on alternate courses of treatment; modify lifestyle to maintain functioning and reduce health declines, etc. [31,32,33]. In other words, the literature suggests that

**H2.**  *Higher levels of patient activation can promote better self-care skills and likewise, better health outcomes.*

Therefore, the broad hypothesis emanating from the above discussion would be that:

**H3.**  *High-quality provider-patient/family communication is associated with higher levels of patient/family activation, which, in turn, is associated with better self-management effectiveness in children with asthma (e.g., higher AAP adherence; higher symptom control; lower ED/inpatient visits; lower outpatient-sick/urgent care visits).*

Future studies seeking to test such hypotheses in turn, would generate empirical evidence on the relationships among a comprehensive collection of variables impacting pediatric asthma self-management effectiveness, which, in turn, would provide a foundation for developing tools and resources for providers to measure and improve self-management effectiveness in children with asthma.

### 1.4. Aims of This Paper

Overall, the above discussion suggests that efforts to identify existing, validated measures of the key constructs and variables in the framework (summarized in Figure 1), might be a productive next step in informing future research involving hypotheses similar to the ones discussed above. Future research of this nature, in turn, would serve the broader purpose of creating a foundation for developing resources & tools for providers to: (i) measure self-management effectiveness of pediatric asthma; and (ii) improve asthma care, by identifying strategies and developing interventions to promote ideal self-management and optimal healthcare use in children with asthma. Correspondingly, the aims of this paper are two-fold:

1) Conduct an integrative review of the literature to identify existing, validated measures of the key constructs and variables in the framework (summarized in Figure 1), with a view to operationalizing the framework.
2) Discuss implications of the operationalized framework for practice, policy, and research related to asthma management.

### 1.5. Special Relevance of the Holistic Framework to Pediatric Asthma

While the operationalized holistic framework on self-management effectiveness would be relevant to all asthma populations (and at a broader level, to chronic disease populations), this review seeks to highlight its special relevance to pediatric asthma populations. The operationalized framework represents an unprecedented opportunity to measure and improve self-management effectiveness of pediatric asthma, since the framework’s key constructs lend themselves more easily and comprehensively to measurement and evaluation in the context of pediatric asthma. This is owing to the existence of strong infrastructural supports (checks and balances) at the societal level, for pediatric asthma management, including family (parental) support, daycare and school support (at the community level), more systematic support at the health system (organizational) level, and strong (environmental) support from social work and public health agencies. There is a higher stake attached to costs of pediatric asthma at the societal level, owing to the “double-whammy” potential for adverse impacts on both the parent/family and the child (e.g., missed work days coupled with missed school days). Given the increased vigilance and societal infrastructure for pediatric asthma management, this review seeks to emphasize the opportunity provided by this framework to make significant contribution to this literature, through future empirical work (that could be spurred by the operationalized framework).

Moreover, highlighting the relevance of the operationalized framework to pediatric asthma, can serve to directly enhance its applicability to adult asthma, since any research efforts utilizing the framework are likely to involve a substantial amount of data collection from both the parent (adult) and child on each of the key constructs. For example, provider-patient communication, patient activation, and medication adherence/symptom control, could (and should) all be assessed from the perspective of both the parent and the child. This implies that the “parent version” of many of these existing validated measures may be easily adaptable to the adult asthma management context.

Importantly, simply because some of the existing validated measures of the key constructs (identified through this review) have not yet been validated with pediatric populations, does not make the operationalized framework any less applicable to pediatric asthma. In fact, the identification of existing validated measures for the key constructs can help by being directly applicable to pediatric asthma care. For example, existing measures of medication adherence are already being administered to both adult and child/adolescent patient populations; and this approach could be extended to patient-reported measures of provider-patient communication as well. On the other hand, the identification of existing validated measures could also help by creating viable opportunities to specifically validate those measures in the pediatric asthma context.

To this effect, this review makes a unique contribution to the asthma management literature by indicating (in Section 2), which measures (of the key constructs) identified through the literature review have already been validated in the pediatric asthma context; and which ones still need to undergo validation with pediatric populations. In summary, the operationalized framework has potential to make significant contributions to pediatric asthma management research and practice, in addition to being relevant to adult asthma and chronic disease management, at a broader level.

## 2. Methodology for the Integrative Review

This paper performs an integrative review of the asthma management literature to address the stated first aim of this paper, i.e., to identify existing, validated measures of the key constructs in the holistic framework for assessing self-management effectiveness in pediatric asthma, with a view to operationalization. Owing to variation in the maturity of the literature streams associated with the measurement of the three key constructs in the framework, a variety of approaches are needed to conduct the integrative review of the literature. To explain, the three broad literature streams that need to be reviewed in accordance with the key constructs of the holistic framework are as follows:

- Literature on measures of provider-patient/family communication (stream #1);
- Literature on measures of patient/family activation (stream #2); and
- Literature on measures of self-management effectiveness of pediatric asthma (stream #3).

To elaborate on the earlier statement related to variation in maturity of these literature streams, it would be important to consider that the literature associated with measuring provider-patient/family communication (stream #1) may be relatively more mature or advanced compared to the literature associated with measuring patient/family activation (stream #2) and the literature associated with measuring self-management effectiveness (stream #3).

The more advanced state of stream #1, may be attributed to substantial interest in measuring the effectiveness of physician-patient communication since the early 1980s, which, in turn, could be ascribed to increasing awareness among physicians, patients, and researchers alike, that effective physician-patient communication may be foundational for achieving desired health outcomes [34]. The advanced state of stream #1 is evidenced by the fact that this body of literature already encompasses multiple systematic reviews of “measures of physician-patient communication,” which, in turn, are discussed in detail in the forthcoming sections. On the other hand, the literature on measuring patient/family activation (stream #2) is relatively contemporary, with origins tracing back to the early 2000s when the Patient Activation Measure (PAM) began to be developed.

With respect to stream #3, in the absence of a holistic framework for measuring self-management effectiveness in children with asthma (discussed earlier), the identification of measures of this complex construct would be best accomplished through a review of the literature associated with measurement of both the “intermediate outcomes” and the “primary outcomes” of self-management effectiveness, outlined in the framework. As discussed earlier, “intermediate outcomes” of self-management effectiveness would encompass two key indicators of AAP adherence, i.e., “medication adherence” and “environmental trigger avoidance.” On the other hand, “primary outcomes” of self-management effectiveness include “asthma symptom control” and “healthcare utilization” for pediatric asthma. When considering all intermediate and primary outcome measures, it becomes apparent that only the two intermediate outcomes (i.e., medication adherence, environmental trigger avoidance) and the first primary outcome (i.e., asthma symptom control), are constructs that would need to be measured through validated instruments. The second primary outcome, i.e., healthcare use, would be a straightforward quantitative measure of healthcare encounters for pediatric asthma in any given research endeavor. In other words, the quest for validated measures would apply only to medication adherence, environmental trigger avoidance and asthma symptom control. This, in turn, translates to a review of the following three sub-streams within stream#3, to identify existing validated measures of self-management effectiveness, in children with asthma:

- Literature on measures of medication adherence (stream #3a);
- Literature on measures of environmental trigger avoidance (stream #3b); and
- Literature on measures of asthma symptom control (stream #3c).

Given the variation in the maturity and intensity of streams #1-#3 and the need to break down stream #3 into sub streams #3a, #3b, and #3c, a variety of approaches will be used to conduct the integrative review in this paper, as summarized in Table 1.

To further elaborate on the information presented in Table 1, the integrative review of the literature on “measures of physician-patient communication,” i.e., stream #1, would be accomplished through a review of all existing systematic reviews of measures of physician-patient communication available in the literature (i.e., a “review of systematic reviews”). On the other hand, the integrative review of streams #2, #3a, #3b, and #3c would be accomplished through keyword searches of PubMed/NCBI (National Center for Biotechnology Information) archives. The process used to review each of the five literature streams (#1, #2, #3a, #3b, and #3c), including the search terms and inclusion/exclusion criteria used to identify a final set of articles for review, is described in Section 2.1, Section 2.2, Section 2.3, Section 2.4 and Section 2.5 below.

### 2.1. Review Approach for Stream #1: Measures of Provider-Patient Communication

Effective communication with healthcare providers has been found to be relevant for patients’ physical and psychological health outcomes as well as treatment adherence. However, the validity of the findings depends on the quality of the applied measures. While a keyword search for “provider-patient communication” or “patient-provider communication” yielded 2165 results on PubMed, narrowing the search down to “patient-provider communication” or “provider-patient communication” and “measurement,” reduced it to 71 results. A screening of the article abstracts however, revealed none of the works specifically involved developing, validating or utilizing “measures” of provider-patient communication. Instead, they pertained to measuring a variety of patient-reported outcomes, including health related quality of life, and broader topics such as the patient-provider relationship.

On the other hand, the search terms “physician-patient communication” or “patient-physician communication” yielded a whopping 27,610 results, and narrowing it to down to “patient-physician communication” or “physician-patient communication” and “measurement,” produced 846 results. This indicates that the preponderance of the work pertaining to measures of “provider-patient” communication exists under the nomenclature of “physician-patient” communication measures.

Of the 846 results, there were 3 “systematic reviews” of measures of physician-patient communication, spanning the past three decades. Adding “asthma” to the aforementioned search terms brought results down to 4, none of which included reviews of measures; and further adding “pediatric” to the search terms reduced it to zero results.

In other words, there have been no measures (no reviews of measures) of physician-patient communication developed specifically for asthma or pediatric asthma. Correspondingly, any research endeavors seeking to evaluate physician-patient communication in the context of pediatric asthma management, would need to apply a “generic” validated measure of physician-patient communication to the context of pediatric asthma care, which, in turn, could provide an opportunity to validate the measure in the context of pediatric asthma. Since the purpose of this review is to identify existing validated measures for the key constructs in the framework, including provider-patient communication, the potential to leverage findings from existing systematic reviews of measures of physician-patient communication, presents an ideal opportunity. Correspondingly, this paper reviews the findings from the following three existing systematic reviews of measures of physician-patient communication, to identify existing validated measures for recommendation: (1) Ong et al. [34]; (2) Boon and Stewart [35]; and (3) Zill et al. [36]. Findings from this review of systematic reviews, are discussed in Section 3.1.

### 2.2. Review Approach for Stream #2: Measures of Patient Activation

The EPR-3 guidelines emphasize the creation of a provider-patient partnership to enable patients/families to monitor and take control of their asthma, so that treatment can be adjusted as needed. This suggests that it may be useful to identify measures of patient empowerment under the broader key construct of self-agency. However, a keyword search on “patient empowerment” AND “measurement” on PubMed, yielded a mere 38 results, and further screening of the abstracts revealed that none of these works involved developing, validating or utilizing “measures of patient empowerment.” This could be explained by the fact that the measurement of the broad concept of patient empowerment, has remained elusive. On the other hand, many of the works resulting from the aforementioned search, utilized validated measures of “patient activation,” e.g., the Patient Activation Measure (PAM), to assess patient empowerment; the argument being, that patient activation, or patient action towards self-care skills is a reflection of patient empowerment. In fact, the fourth stage in the 4-stage developmental model of activation intrinsic to the PAM, has been put forth by the literature, to be a measure of patient empowerment [31].

These gleanings helped refresh the search terms to “patient activation” and “measurement.” Of the 63 search results, only 23 were empirical studies that involved using a measure of patient activation. Of the 23 studies 19 (82%) utilized the PAM to measure patient activation. In the remaining studies, “patient activation” was defined in terms of “patient engagement,” which, in turn, was measured using other instruments, like the Community Health Activity Index (CHAI) or the Person Engagement Index (PEI). It would be relevant to note that adding *“pediatric”* to the search terms (i.e., “patient activation” and “measurement” and “pediatric”) reduced the results from 63 to 3 results, and further adding *“asthma”* to the search terms returned zero results. In other words, there have been no measures of patient activation developed specifically for asthma or pediatric asthma. However, one of the 3 results returned under the search for *“pediatric,”* utilized a parent version of the PAM (P-PAM) to measure “parent activation” in the context of pediatric primary care outcomes for vulnerable children [37]. Overall, similar to the search on provider-patient communication, the searches related to patient activation revealed that any research endeavors seeking to evaluate patient activation in the context of pediatric asthma management, would need to apply a “generic” validated measure of patient activation to the context of pediatric asthma care, which, in turn, could provide an opportunity to validate the measure in the specific context of pediatric asthma care.

Given the domination of PAM as a measure of patient activation in the empirical literature, the renewed focus of the literature search was on understanding the development and validation process for PAM, to authenticate its suitability for use as a measure of the key construct of patient activation in the holistic framework. To this effect, the search terms *“Patient Activation Measure” and “validation”* retuned 29 results, a majority of which involved validating PAM in a variety of patient populations, patient conditions, and geographic regions, include Brazil, Norway, Korea, and Italy. Of the 29 results, only three focused on describing the development and validation of the “generic” Patient Activation Measure (PAM) [31,38,39]. These three articles are reviewed in Section 3.2, to determine the appropriateness of using PAM as a measure of patient activation in the holistic framework described in this review.

### 2.3. Review Approach for Stream #3a: Measures of Medication Adherence

Poor medication adherence in children with asthma is known to be a major cause of acute asthma exacerbation and poor symptom control. Correspondingly, identifying effective ways to measure and monitor medication adherence is considered a key component of pediatric asthma management. While the search terms “medication adherence” and “measurement” on PubMed resulted in a whopping 10,886 results on PubMed, narrowing the search to “medication adherence” and “measurement” and “pediatric” reduced it to 30 results; and adding “asthma” to the search brought it down to just two results. One of the two results however, turned out to be an integrative review of medication adherence monitoring tools for children and adolescents with asthma published in fall 2018, making it highly relevant to this review effort [40]. Section 3.3 discusses key gleanings from the selected article.

### 2.4. Review Approach for Stream #3b: Measures of Environmental Trigger Avoidance

Indoor environmental exposures, particularly allergens and pollutants, are major contributors to asthma morbidity in children; and environmental control practices aimed at reducing these exposures are an integral component of asthma management. While the search terms “environmental trigger avoidance” and “measurement” yielded no results on PubMed, the terms “environmental control practices” and “asthma” yielded 10 results. Further adding “pediatric” to the search terms brought it down to two results, and one of these works sought to measure comprehensive use of environmental control practices (ECPs) as a key component of preventive pediatric asthma care, making it highly relevant to this integrative review [41]. Section 3.3 discusses key gleanings from the selected article.

### 2.5. Review Approach for Stream #3c: Measures of Asthma Symptom Control

Regular assessment and monitoring of symptoms are key components of recommended care for pediatric asthma management. A keyword search of “asthma control test” and “pediatric” on PubMed yielded 138 results. A narrower search for reviews of asthma control assessment tools in pediatric care (using the terms “asthma control” and “assessment” and “pediatric” and “review”) yielded 32 results. While these articles addressed multiple aspects of pediatric asthma symptom control across various settings of care and geographic regions, one recently published article, sought to provide a comprehensive review of asthma control assessment tools for both adult and pediatric asthma, making it highly relevant to this integrative review [42]. Section 3.3 discusses key gleanings from the selected article.

The next section (Section 3) describes the essential findings from all aforementioned integrative review efforts. The description of findings in turn, culminates in the development of a *matrix* linking the Constructs and Recommended Validated Measures emanating from this review. This *matrix,* summarized in Table 2, therefore, serves as the culminating deliverable of this review, in operationalizing the framework summarized in Figure 1. The final sections of the paper discuss key implications of the *matrix* (Table 2) for asthma management research and practice.

## 3. Key Findings from the Integrative Review

This subsection describes key findings from the integrative literature review conducted to identify existing, validated measures of the key constructs encompassed in the holistic framework, i.e., (1) measures of provider-patient/family communication; (2) measures of patient/family activation; (3) measures of self-management effectiveness.

### 3.1. Findings from Review of Measures of Provider-Patient Communication (Stream #1)

Since the importance of physician-patient communication has been increasingly recognized, a considerable number of instruments seeking to measure physicians’ communication skills have been developed over time. As indicated in Section 2.1, the literature search conducted on PubMed revealed three systematic reviews of measures of physician-patient communication, spanning three decades: (1) Ong et al. [34]; (2) Boon and Stewart [35] and (3) Zill et al. [36].

Ong et al. addressed disparate topics related to physician-patient communication. They included a chapter on the different purposes of medical communication, reviewed specific communication behaviors and their influences on patient outcomes, and performed an analysis on physician-patient communication. For the latter, they presented a brief overview of different measures of physician-patient communication. However, they did not evaluate the psychometric properties of the instruments [34].

A further comprehensive review, conducted by Boon and Stewart, compared measures of physician-patient communication published between 1986 and 1996. They found 44 instruments which they reviewed for reliability and validity [35]. However, these instruments were primarily developed for use in medical education settings, and the review included manuals of measures without a validation study published in a peer-reviewed journal.

The more recent systematic review conducted by Zill et al. provides the most current comparison and evaluation of existing instruments of physician-patient communication based on clearly defined quality criteria [36]. This systematic review (PROSPERO code: CRD42013005687) provides a comprehensive overview of generic measures of physician-patient communication. It evaluates the quality of design, methodology, and reporting of studies that present psychometric properties of measures, using the COnsensus based Standards for the selection of health status Measurement INtruments (COSMIN) checklist [43,44]. It then proceeds to determine the quality of the psychometric properties of identified measures using the criteria specified by Terwee and colleagues [45]. These criteria include psychometric properties of content validity, internal consistency, criterion validity, construct validity, reproducibility (agreement and reliability), responsiveness, floor and ceiling effects, and interpretability. Data from 25 studies on 20 measures of physician-patient communication were included in this review. More than half of these studies were conducted in the United States or Europe. Study settings included outpatient practices, outpatient units of hospitals, and medical centers. Of the 20 measures of physician-patient communication included in this review, 11 measures used observer-rating systems; five measures were patient-reported; two measures used both physician- and patient-reports; one measure used the physician’s rating solely; and a last measure was a computer-based analysis.

When combining the ratings of the studies on the COSMIN and Terwee et al. criteria, the best results were noted for studies on the following measures: the Patient-centered Behavior Coding Instrument (PBCI), the SEGUE framework, and the Questionnaire on Quality of Physician-Patient Interaction (QQPPI) [36]. Each achieved at least two excellent or good ratings by COSMIN and two positive ratings by Terwee et al.’s criteria. Two of the three measures that fared best in the Zill et al. review (PBCI and the SEGUE framework) are observer-rated measures of physician-patient-communication, while the third (QQPPI) is a patient-reported measure [46,47,48]. None of the physician-reported measures or the patient-and-physician reported measures fared well on the review. While observer rating of recorded consultations is considered the gold standard, they tend to be too complex and time-consuming to implement in routine care. Physician self-report scales, in turn, are prone to bias since they reflect physicians’ subjective perception of their own performance. Therefore, *patient-reported measures* constitute a good compromise in terms of reliability and feasibility, and are increasingly being used for improving quality of care [36]. The results of the systematic review by Zill et al. [36] are highly relevant to researchers seeking to identify suitable validated measures of physician-patient communication for use in research studies.

Future studies seeking to validate the framework (Figure 1) and test hypotheses related to relationships among key constructs and variables in the framework, would benefit most from a *patient-reported measure* of physician-patient communication, given that data on the other key constructs in the framework summarized in Figure 1, i.e., patient activation and self-management effectiveness in pediatric asthma would also need to be collected from the perspective of patients/families. In the case of pediatric asthma, all of the key constructs and variables in the framework could be assessed from the perspective of both the parent and the patient (e.g., child >9 and <18 years of age).

Therefore, based on the results of the systematic review by Zill et al. [36], the QQPPI emerges as the frontrunner for use as a validated patient-reported measure of physician-patient communication. QQPPI is a 14-item patient self-reporting instrument that allows assessment of the quality of physician–patient interactions during routine outpatient care. It can also be used to evaluate physician communication training programs for educational purposes [48,49,50]. The development and validation of the QQPPI has been described in multiple peer-reviewed publications [48,49,50]. Because QQPPI scores are independent of patient characteristics (i.e., age, gender, education) and are not confounded by social desirability or health status, the instrument has been found to be superior in identifying genuine determinants of physician-patient interactions. In summary, the QQPPI has been found to be a valid and reliable patient self-reporting instrument, allowing the efficient assessment of the quality of the physician-patient interaction in the ambulatory care setting. In addition, the QQPPI has been found to have the potential to discriminate among the communication performances of individual physicians.

Importantly, a closer review of the QQPPI reveals that the instrument incorporates the key functions of high-quality physician-to-patient communication referenced in the Street et al. model (i.e., information sharing, emotional support, managing uncertainty, creating relationships, decision making, and self-management). In other words, QQPPI is an existing validated instrument that could be used to apply the Street et al. framework for evaluating the quality of physician-patient communication. This also implies that the QQPPI could be supplemented with other validated instruments for measuring immediate outcomes of physician-patient communication, also referenced in the Street et al. model, including (1) the Asthma Self-Management Questionnaire (ASMQ) to measure “*Understanding*”; (2) Trust in Physician Scale (TPS) to measure “*Trust*”; (3) the patient-provider agreement instrument to measure “*Agreement*”; (4) the Perceived Involvement in Care Scale (PICS), to measure “*Involvement*”; and (5) a single-item instrument to measure “*Motivation*” [51,52,53,54,55].

### 3.2. Findings from Review of Measures of Patient/Family Activation (Stream #2)

As mentioned in Section 2.2, the emphasis of the literature search pertaining to the key construct of self-agency, was on understanding the development and validation of a measure that almost exclusively dominated the empirical literature on patient activation, i.e., the PAM. As discussed, the review returned three articles that focused on the development and validation of the generic PAM measure, which was of particular relevance to this review [31,38,39].

Although patient activation is a central concept in both the consumer-driven health care framework and chronic illness care models, it was conceptually and empirically underdeveloped prior to the development and validation of PAM by Hibbard et al., in the early 2000s [31]. Since then, PAM has gained widespread recognition as a valid and reliable instrument for measuring patient activation. Prior to the development of PAM, there were limited methods available for assessing different aspects of activation, such as health locus of control, self-efficacy in self-managing behaviors, and readiness to change health-related behaviors; however, these measures tend to focus on the prediction of a single behavior [56,57,58,59]. PAM, on the other hand, enables assessment of a broad range of elements reflected in patient activation, including the knowledge, skills, beliefs, and behaviors needed to manage a chronic illness. Those who are activated, believe that patients have important roles to play in maintaining their own health. They know how to manage their condition, maintain functioning, and prevent health declines, and they have the skills and the behavioral repertoire to do so.

A review of the articles describing the development and validation of the generic PAM measure revealed that Hibbard et al. used a multi-stage approach to develop and validate the PAM, using several national probability samples of patients [31,38,39]. Essentially, the PAM was developed in four stages described below:

*Stage 1*. Conceptually defining activation first involved a literature review, systematic consultation with experts using a ‘‘consensus method,’’ and consultation with individuals with chronic disease, using focus groups. The review of the literature indicated that patients who are able to: (1) self-manage symptoms/problems, (2) engage in activities that maintain functioning and reduce health declines, (3) be involved in treatment and diagnostic choices, (4) collaborate with providers, (5) select providers and organizations based on performance or quality, and (6) navigate the health care system, are likely to have better health outcomes. These six domains were used as a starting point for an expert consensus process and for patient focus groups.

*Stage 2*. Preliminary scale development began by building on the domains identified in Stage 1 and operationalizing them with survey items within each domain. Steps included generating, refining, and testing a large item pool. Rasch psychometric methods were used to develop the scale and test the preliminary measure’s psychometric properties.

*Stage 3*. The next stage involved exploring the possibility of extending the range of the measure, refining the response categories, and testing whether the measure could be used with respondents who had no chronic illnesses.

*Stage 4.* In the fourth and final stage, a national probability sample was used to assess the performance of the measure across different subsamples in the population, and to assess the construct validity of the measure.

Following the comprehensive multi-stage development and validation process, PAM has been found to be a valid, highly reliable, unidimensional, probabilistic scale that reflects a *developmental model of patient activation, in four stages*: (1) believing the patient role is important, (2) having the confidence and knowledge necessary to take action, (3) taking action to maintain and improve one’s health, and (4) staying the course even under stress. PAM has been found to have good psychometric properties, indicating that it can be used at the individual patient level to tailor intervention and assess changes.

### 3.3. Findings from Review of Measures of Self-Management Effectiveness (Streams #3a, #3b, and #3c)

According to the framework summarized in Figure 1, *self-management effectiveness* of pediatric asthma can be measured in terms of both intermediate outcomes and primary (health) outcomes.

#### 3.3.1. Intermediate Outcomes

These include measures of Asthma-Action Plan (AAP) adherence, which, in turn, includes measures of: 1) medication adherence; and 2) environmental trigger avoidance:

*Medication Adherence* (stream #3a): As discussed in Section 2.3, the review process for measures of medication adherence for pediatric asthma, identified a recently published article that sought to synthesize the literature on medication adherence measurement and monitoring tools for pediatric asthma [40]. The review sought to critique a comprehensive collection of existing subjective and objective methods for measuring medication adherence, including validated self-report scales, under subjective methods. Self-report instruments could be completed by the parent and/or the child, depending on their development and validation. The most frequently used self-report instruments to measure medication adherence in asthma are the Morisky scale [60] and the Medicine Adherence Report Scale—Asthma (MARS-A) [61].

The Morisky scale, which was originally developed as a 4-item questionnaire for hypertension medication adherence was recently developed into a predictive 8-item questionnaire with dichotomous answers except the final answer which was is a five-point Likert scale [62]. The Morisky scale has been used in a variety of health conditions, including asthma. The studies that have used the Morisky scale were conducted in mixed populations which included both children and adults. In 2017, the Morisky scale was developed and validated for use in asthma as an 8-item questionnaire (Morisky Medication Adherence Scale, MMAS-8), in patients over 12 years old. In these studies, the questionnaire was found to correlate well with asthma control and quality of life [40,62].

The MARS was originally developed and validated in multiple disease populations including asthma. It consists of nine items, all scored on a 5-point Likert scale, and it has been adapted for the asthmatic population specifically to address adherence to inhaled corticosteroids. The MARS-A, a 10-item scale, was validated in adult patients with asthma and has been found, in research studies, to be moderately correlated with objective electronic monitoring data. However, in children it is often the parents who complete the questionnaire on behalf of the child and both the MARS-A and the MARS-5, a shortened version, have been found to be inaccurate in children when administered in clinical practice and compared to objective electronic monitoring data [40,61].

The review emphasizes the importance of supplementing subjective data on medication adherence captured through validated self-report scales like MMAS and MARS, with objective data on medication adherence, to boost the accuracy of measurement. Objective measures of medication adherence would include prescription data (fill rates), weighing inhaler canisters, nurse home visits, electronic monitoring device data for inhalers, using biomarkers for adherence, and integrating digital technologies to monitor adherence. In summary, based on the comprehensive review of existing validated self-report scales for medication adherence, the MMAS comes across as being more suitable (compared to MARS) for measuring medication adherence in children with asthma. Correspondingly, the MMAS would be recommended for measuring medication adherence within the holistic framework in this review. Depending on the nature of the research study emanating from the holistic framework, data from MMAS could be supplemented with objective data as available, to improve the accuracy of the medication adherence measure.

*Environmental trigger avoidance* (stream #3b): As discussed in Section 2.4, the review process for measures of environmental trigger avoidance for pediatric asthma, identified an article that sought to examine the association between preventive pediatric asthma care and comprehensive use of Environmental Control Practices (ECPs) among children with asthma [41]. This study used the Environmental Section of the National Asthma Survey (NAS) to assess comprehensive ECP use. This validated instrument (NAS) includes a comprehensive scale to measure use of eight ECPs: by children and their families: (i) air filter, (ii) dehumidifier, (iii) mattress cover, (iv) pillow cover, (v) pet avoidance, (vi) smoke avoidance, (vii) removing carpets, and (viii) washing sheets in hot water. In summary, the literature suggests that the Environmental Section of the NAS would be a comprehensive existing validated tool for measuring environmental trigger avoidance for pediatric asthma, within the holistic framework in this review [63,64].

In any research endeavor, data obtained from validated self-report instruments on medication adherence and environmental trigger avoidance, could be supplemented with—or verified through—secondary data sources, as applicable. For example, self-reported data on medication adherence (captured through MMAS) could be verified through secondary data from medical records on prescription refills from the patient’s pharmacy. On the other hand, self-reported data pertaining to environmental trigger controls (captured through NAS questions), could be verified through secondary data related to the age, structure, and pricing characteristics of homes, as applicable, obtained from public online housing databases (such as Zillow.com and Realtor.com).

#### 3.3.2. Primary (Health) Outcomes

Reflecting national guidance for assessing pediatric asthma control, primary outcomes would include validated measures of asthma symptom control, in addition to hard outcomes measures of healthcare use for pediatric asthma.

*Asthma symptom control* (stream #3c). As discussed in Section 2.5, the review process for measures of pediatric asthma symptom control identified a recently published comprehensive article on asthma control assessment tools [42]. This review examined tools that have been validated, found to have established psychometric properties, and have been extensively studied in terms of their ability to reflect the overall status of asthma control. The tools included are the Asthma Control Test (ACT), Child Asthma Control Test (cACT), Asthma Control Questionnaire (ACQ), Asthma Therapy Assessment Questionnaire (ATAQ), and Lara Asthma Symptom Scale (LASS). Among these, the cACT and the ACT were the only asthma symptom control assessment tools that have been applied to pediatric asthma, with cACT being applicable to children 4–11 years old and ACT being applicable to children 12–17 years old [65,66,67].

As described in the review, the cACT has been validated more than any other asthma control assessment tool for children with asthma, and, therefore, it has been designated as a core outcome for National Institutes of Health (NIH)-initiated participant characterization and for observational studies. The cACT was developed in 2006 to assess asthma control in 4–11 y old children. The cACT is a self-administered tool that integrates the child’s and the child’s caregiver’s perspectives on asthma control over the previous 4 weeks. The cACT is composed of seven questions (four child-reported and three caregiver-reported). The child-reported questions, rated on 4-point Likert scale, include daytime and activity limitation due to asthma symptoms, nocturnal awakenings due to asthma, and self-perceptions of asthma; the caregiver-reported questions, rated on 6-point Likert scale, include asking about the child’s daytime and nocturnal symptoms. The responses summed to an overall score that ranges from 0 (poor control of asthma) to 27 (complete control of asthma) [67].

Similar to the cACT, the overall validity of the ACT has been assessed more than any other asthma control assessment tool. Thus, the ACT has been designated as a core measure for NIH-initiated clinical research in adults. The ACT is a multidimensional, standardized, and validated tool and the most widely used tool for assessing asthma control in patients with asthma, older than 12 years. Similar to most asthma assessment tools, the ACT quantifies asthma control as a continuous variable and provides a numeric value to distinguish between controlled and uncontrolled asthma. The ACT is a patient-centered/completed questionnaire that recalls the patient’s experience of 5 items: asthma symptoms (nocturnal and daytime), the use of rescue medications, the effect of asthma on daily functioning, and the patient’s perception of asthma control over the previous 4 weeks. Each item includes five response options corresponding to a 5-point Likert-type rating scale. Subsequently, responses for each of the five items are summed to yield a score ranging from 5 (poor asthma control) to 25 (complete asthma control [65,66]. In summary, the literature suggests that the cACT and the ACT would be appropriate existing validated measures to use for assessing child asthma symptom control, within the holistic framework described in this review [42,65,66,67].

*Healthcare use for pediatric asthma***.** As discussed earlier, since healthcare use can be measured as a straightforward count of healthcare encounters in any research endeavor, it does not require a validated measure, identified through literature review. Nevertheless, it would be important to outline a basic measure for healthcare use for pediatric asthma in this review, for the purpose of (1) completing the operationalization of the holistic framework and (2) offering a basic quantitative measure of healthcare use for pediatric asthma that could be utilized in any research endeavor emanating from the holistic framework.

Essentially, healthcare use for pediatric asthma could be defined as the patient/family’s aggregate healthcare encounters for pediatric asthma over a period of time (defined for any given research effort). This, in turn, may include:

- *Total number of encounters for pediatric asthma care,* i.e., aggregate of outpatient visits (routine and sick), inpatient admissions (general admission and PICU), ED and urgent care visits for pediatric asthma care over a period of time, adjusted by the patient’s severity of illness.
- *Total number of “non-revisits” for pediatric asthma outpatient care* i.e., patient/families who do not return even once for outpatient care, over a six-month period following an initial visit, adjusted by the patient’s severity of illness.
- *Total number of “no-shows” for pediatric asthma outpatient care*, i.e., patient/families who do not show up for scheduled appointments for pediatric asthma outpatient care, adjusted by the patient’s severity of illness.

### 3.4. Drawing upon Established Measures for Other Socio-Ecological (Control) Variables in the Framework

Similar to the inclusion of measures for healthcare use of pediatric asthma, it is necessary to include measures for the other socioecological (moderating and mediating) variables encompassed in the framework, in order to complete the operationalization of the holistic framework. While many of these socio-ecological variables would require measures based on straightforward counts and percentages in any research endeavor (similar to healthcare use), some of the socio-ecological variables exist in the form of constructs, requiring validating instruments for measurement. Although a detailed literature review to identify validated measures for each construct-based socio-ecological variable is beyond the scope of this paper, it is possible to identify nationally validated, established, and widely-used measures for these constructs, without a detailed integrative review. The main purpose of this approach would be to provide a complete operationalization of the holistic framework, in keeping with the stated first aim of this paper.

The measures identified for each socio-ecological variable encompassed in the framework are outlined below:

○ *Individual demographic characteristics* (e.g., age, gender, race, school attended and grade level, names of pharmacy and primary care physician, family history of asthma) and primary caregiver’s demographic characteristics (age, race, income, employment, insurance status, tobacco use, zip code, etc.) could be captured through patient surveys, and further supplemented/verified through medical record review in any research endeavor. Similarly, the health literacy of children with asthma and their families could be assessed using widely-used existing validated instruments: (1) Rapid Estimate of Adult Literacy in Medicine (REALM) or (Rapid Estimate of Adolescent Literacy in Medicine (REALM-Teen), (2) Single Item Literacy Screener (SILS), and (3) Newest Vital Sign (NVS) [68,69,70,71,72].
○ *Individual biological variables*, such as asthma severity, could be defined using the EPR-3 asthma severity categorization framework, which, in turn, could be captured through patient surveys, and further verified through medical record review in any research endeavor [5].
○ *Interpersonal and socio-economic variables* would be assessed using validated instruments like the Cohen Social Support Index and Holmes and Rahe Stress Scale; in addition to fact- based questions for families, e.g.: *Has your insurance company denied coverage for your child’s asthma control or relief medications? Does your child live in: (1) one home; or (2) more than one home?* [73,74,75].
○ Health system variables. In addition to the QQPPI measures of physician-patient communication, health system (organizational) factors influencing asthma management could be assessed using validated National Asthma Survey (NAS) questions on *Self-Management Education Received from Providers* [63]. Examples from this survey include: *Has your child’s primary care (or clinic doctor) ever: (1) shown you how to use your child’s inhaler? (2) taught you how to use your child’s peak flow meter to adjust his/her daily medications?*
○ *Community-level variables*, such as school support for asthma, could be assessed using validated questions from the CDC Healthy Schools initiative [76]. For example*: (*1*) Does your school/daycare have a copy of your child’s AAP?* (2) *Does someone in your school/daycare give your child his/her controller medication?*
○ *Environmental variables* in turn, could be assessed using the “Environment Section” of the validated National Asthma Survey (NAS) [63]. A key relevant feature of this section within the NAS, is that it includes questions to distinguish between “environmental risk factors” beyond the control of patient/family (e.g., *how old is your home?);* and “environmental trigger control,” or actions within the control of patient/family (e.g., *does anyone in your household smoke?).*

### 3.5. Operationalizing the Holistic Framework

The integrative review effort described in this paper, serves to identify existing, validated *measures* of key constructs in the holistic framework for assessing self-management effectiveness in pediatric asthma, which, in turn, could be used to organize next steps for research in asthma management. The findings described in Section 3, help to recommend existing, validated measures of key constructs to enable operationalization of the framework for future research targeted towards examining the relationships among key constructs in the framework. Table 2 provides a matrix linking the Constructs and Recommended Measures from this integrative review effort, as a point of culmination for this operationalization. It would be relevant to note that the matrix incorporates recommended measures emanating from the review effort (described in Section 3.1, Section 3.2 and Section 3.3), as well as the generic established measures for the socio-ecological factors discussed in Section 3.4.

## 4. Discussion

### 4.1. Implications for Future Research

This review makes contributions to not only to the asthma management literature, but also to the broader literature on chronic disease management in children, by helping to operationalize a holistic framework for measuring self-management effectiveness in pediatric asthma. Essentially, an operationalized framework of this nature would be significant in explicating the impact of provider-patient communication on self-management effectiveness, through the pathway of patient/family activation (self-agency). The current state-of-the-art literature on pathways by which physician-patient communications impact self-management and outcomes, is the model put forth by Street et al., as discussed [22]. However, this is only a conceptual model, with substantial potential for modification through empirical research. While a recent study (Young et al., [77]) sought to examine the relationship between physician-patient communication and self-care skills for asthma, using the Street et al. model, it was limited by: (1) not utilizing validated measures of physician-patient communication, (2) defining self-care skills solely in terms of medication adherence (rather than AAP adherence as a whole), (3) lack of correlation with patient health outcomes, and (4) having a retrospective rather than prospective study design [77].

By contrast, the operationalization of the framework described in this paper could provide a foundation for groundbreaking prospective studies seeking to examine relationships among quality of provider-patient/family communication, patient/family activation, and self-management effectiveness in pediatric asthma, with the latter defined in terms of both intermediate outcomes (AAP adherence) and primary outcomes (symptom control and healthcare use). Importantly, such research endeavors could utilize the robust existing validated instruments (identified in this review), for measuring the key constructs & variables in the framework. The results of these initiatives, in turn, could pave the way for individual providers and organizations (hospitals/clinics) to measure and improve self-management effectiveness in pediatric asthma.

While the Street et al. model focuses on the impact of physician-patient communication on health outcomes via immediate outcomes like trust, understanding, agreement, involvement, and motivation, a broader limitation is that it does not emphasize the link between *provider-patient communication* and *patient activation*. The latter link is crucial from the perspective of both engaging providers in asthma management and ensuring successful implementation of EPR-3 clinical practice guidelines. Future research could help to overcome this conceptual limitation, by utilizing the holistic framework described in this paper, to investigate the *relationship among provider-patient communication, patient activation, and self-management effectiveness*, using existing, validated measures.

In doing so, not only would future research (based upon this framework) enable assessment of the relationship between provider-patient communication and patient/family activation using validated measures, but it would also facilitate measurement of the immediate outcomes of physician-patient communication put forth by Street et al. This would be possible because, the concept of immediate outcomes of physician-patient communication put forth by Street at al. would fit neatly under “Health System” level of influences, outlined in Figure 1. Importantly, employing a prospective approach to measuring the relationships among key constructs in the framework, including AAP adherence and validated patient-reported outcomes in pediatric asthma, would help to overcome limitations of previous retrospective, cross-sectional, and fragmented approaches to measuring these relationships.

### 4.2. Implications for Practice and Policy

The operationalized framework developed in this paper (and summarized in Figure 1 and Table 2), has the potential to make significant contributions to asthma management practices. For example, data from the CDC on suboptimal adherence to EPR-3 practice guidelines suggests that providers may be playing a more *reactive* role rather than a *proactive* role in promoting asthma self-management [1,7,8,9]. In order to adopt a more proactive role, providers need resources and tools for measuring self-management effectiveness of patients/families with asthma. The operationalized framework developed in this paper provides a foundation for future research directed towards understanding of the relationships among three key constructs: provider-patient communication, patient activation, and self-management effectiveness in pediatric asthma. These results could directly contribute to the development of resources and tools for providers to measure and improve self-management effectiveness in pediatric asthma. Hypothetical examples are provided below.

For example, patients/families with lower health literacy might experience a higher quality of provider-patient/family communication when their encounter includes communication with multiple providers, including an asthma educator. Subsequently, higher quality communication might generate higher levels of patient activation and better self-management effectiveness. Similarly, a majority of younger parents (age <25 years) are likely to hold multiple jobs, and face challenges caring for children with asthma. Children in these homes might be found to exhibit lower levels of AAP adherence due to poor medication adherence stemming from irregular refills of prescription medications or lack of medication administration supervision. Irregular controller medication refills or use may then translate to higher numbers of ED/urgent visits for asthma exacerbations in these children. This group of parents may have also experienced lower quality provider-patient communication and lower levels of patient/family activation, in addition to lower levels of school support for asthma management. Additionally, patients living in homes older than 25 years may have exhibited the lowest adherence to the environmental trigger avoidance component of their AAP, which in turn, could have translated to a higher number of ED visits, urgent care visits, and hospitalizations for pediatric asthma.

In other words, future research of the nature described above, would help generate profiles of patients/families with higher and lower self-management effectiveness which, in turn, would provide insight into health disparities in asthma management within the population being served. Such evidence, in turn, would create a foundation for developing resources & tools for providers to use in measuring self-management effectiveness of pediatric asthma, like for example, tools to rapidly assess patients’ health literacy, AAP adherence, asthma symptom control, and healthcare use since the patient/family’s last healthcare encounter. Importantly, such research endeavors would provide a typology for developing strategies and interventions for promoting ideal self-management and optimal healthcare use in children with asthma.

For example, based on the hypothetical relationships discussed above, providers might infer that lower-literacy patients/families could greatly benefit from additional resources for asthma education from providers, including renewed efforts from subspecialists and primary care physicians to provide self-management education at an appropriate level. Similarly, providers may infer that younger parents could benefit from interventions designed to promote regular refills of prescription medications as well as school support for asthma management. Likewise, patients living in homes with environmental challenges could benefit from collaborative efforts between the hospital (provider) community and the state housing department to promote the use of medically recommended air conditioners for asthma patients living in those homes, as well as other collaborative ventures to improve the overall living condition of those homes.

In addition to these *practice* and *policy* implications for reducing the public health burden of pediatric asthma, the operationalized framework developed in this paper could provide a stepping stone for future *research* endeavors seeking to: (1) develop simple resources and tools (for providers) to measure self-management effectiveness, using both patient-reported outcomes (PROs) and insights gained on relationships among key constructs in the framework, (2) implement community-based interventions (identified from research efforts) to promote optimal self-management and healthcare use among pediatric asthma patients/families; (3) integrate PROs for pediatric asthma into the EHR to promote quality of care and self-management effectiveness, and (4) evaluate the generalizability of results from future endeavors through large-scale replication in outpatient settings in rural and inner-city regions across the U.S. Such future research endeavors would serve a dual purpose of addressing gaps in the asthma literature while tackling the practical challenges of pediatric asthma management. Ultimately, such concerted efforts would have potential to alleviate the public health burden and healthcare costs of pediatric asthma as well as other chronic diseases in the community.

### 4.3. Limitations of the Review

The broad limitation of this integrative review is that it is tailored towards operationalizing the key constructs of a previously developed conceptual framework for assessing self-management effectiveness of pediatric asthma. To this effect, it is guided and shaped by the structure of the previously developed framework, which in turn, limits its ability to be open-ended and systematic in identifying measures of self-management effectiveness. However, the same limitation may be viewed as strength, given the fact that the earlier conceptual framework was developed to promote a holistic measure of self-management effectiveness that takes into account both factors within and beyond the control of patients/families in impacting asthma self-management. In doing so, the framework serves to address a core gap in the asthma management literature, in regard to a holistic measure for self-management effectiveness that providers could leverage to become more engaged in promoting ideal self-management and optimal healthcare use in children with asthma. Existing research on asthma self-management is fragmented and cross-sectional, and greatly limited by the absence of a holistic framework. This review serves the unique purpose of operationalizing a previously developed holistic framework, to create a foundation for empirical research in this area, to ultimately enable provider engagement in asthma management and the successful implementation of EPR-3 national evidence-based practice guidelines for asthma management.

## 5. Conclusions

This paper performs an integrative review of the literature to operationalize the key constructs and variables in a holistic framework for assessing self-management effectiveness in pediatric asthma. In doing so, a foundation is laid for future research seeking to explicate the relationships among provider-patient/family communication, patient activation, and self-management effectiveness in pediatric asthma. The results of such research endeavors have the potential to engage asthma providers in: (1) identifying strategies for promoting ideal self-management and optimal healthcare use at the patient/family level, and (2) developing interventions for promoting asthma self-management effectiveness, at the community level, as applicable. Therefore, in addition to accelerating the adoption of patient-centered care for asthma management, the operationalized framework would have potential to enable successful implementation of EPR-3 clinical practice guidelines for asthma management, and thereby facilitate a focus on “population health management,” by asthma providers, in a new era of value-based reimbursement. This integrative model in turn, would have the potential to positively impact self-management effectiveness in other chronic disease states, in both children and adults.

## Figures and Tables

**Table 1:** Summary of Approaches Used for Integrative Review.

Stream #	Stream Name	Review Approach	Description
Stream #1	Measures of Provider-Patient/Family Communication	Review of Systematic Reviews.	Review of all existing “systematic reviews” of “measures of physician-patient communication,” to identify existing validated measures for recommendation. Details of the review process are described in Section 2.1.
Stream #2	Measures of Patient/Family Activation	Keyword search of stream #2 literature.	Keyword search of PubMed archives with inclusion/exclusion criteria to identify a final set of articles for review. Articles are reviewed with a view to identifying existing validated measures for recommendation. Details of the review process are described in Section 2.2.
Stream #3a	Measures of Medication Adherence	Keyword search of stream #3a literature.	Keyword search of PubMed archives with inclusion/exclusion criteria to identify a final set of articles for review. Articles are reviewed with a view to identifying existing validated measures for recommendation. Details of the review process are described in Section 2.3.
Stream #3b	Measures of Environmental Trigger Avoidance	Keyword search of stream #3b literature.	Keyword search of PubMed archives with inclusion/exclusion criteria to identify a final set of articles for review. Articles are reviewed with a view to identifying existing validated measures for recommendation. Details of the review process are described in Section 2.4.
Stream #3c	Measures of Asthma Symptom Control	Keyword search of stream #3c literature.	Keyword search of PubMed archives with inclusion/exclusion criteria to identify a final set of articles for review. Articles are reviewed with a view to identifying existing validated measures for recommendation. Details of the review process are described in Section 2.5.

**Table 2:** Matrix Linking Key Constructs in Holistic Framework to Recommended Existing Validated Measures.

Construct	Sub-Construct	Variable	Recommended Existing Validated Measure(s)/Instrument(s)/Question(s)
**Socio-Ecological Factors**	Individual Biologic & Demographic Characteristics	Asthma Severity	EPR-3 Asthma Severity Classification (1-Intermittent; 2-Mild-Persistent; 3-Moderate-Persistent; 4-Severe-Persistent)
		Health Literacy	Rapid Estimate of Adult Literacy in Medicine (REALM)
			REALM Teen
			Single-Item Literacy Screener (SILS)
			Newest Vital Sign (NVS)
		Other Demographic characteristics	Miscellaneous validated questions to ascertain demographic characteristics
**Socio-Ecological Factors**	Interpersonal and Socio-economic Risk Factors	Social Support	Cohen Social Support Index
		Stress	Holmes and Rahe Stress Assessment Instrument
**Socio-Ecological Factors**	Health System (Organizational Factors)	Provider-Patient/Family Communication	Questionnaire on Quality of Physician-Patient Communication (QQPPI)
		Provider-Patient/Family Communication of Asthma Action Plan (AAP)	National Asthma Survey (NAS) questions related to asthma self-management education by providers
		Immediate Outcomes of Provider-Patient/Family Communication	Asthma Self-Management Questionnaire (ASMQ); Trust in Provider Scale (TPS); Steigers’s Agreement Instrument; Perceived Involvement in Care Scale (PICS); Riekert Motivation Scale.
**Socio-Ecological Factors**	Community-Level Factors	School Support for Asthma Management	Centers for Disease Control and Prevention (CDC) Healthy Schools Questionnaire
**Socio-Ecological Factors**	Environmental-Level Factors	Indoor Environmental Air Quality	National Asthma Survey (NAS) section on Environmental Risk Factors
**Self-Agency**	Patient Activation	Patient/Family Activation in Asthma Management	Patient Activation Measure (PAM)
**Self-Management Effectiveness of Pediatric Asthma**	Asthma-Action Plan (AAP) Adherence	Adherence to Medication (Intermediate Outcome)	Morisky Medication Adherence Scale (MMAS)
		Adherence to Environmental Trigger Controls (Intermediate Outcome)	National Asthma Survey (NAS) section on Environmental Trigger Control
	Asthma Control	Asthma Symptom Control (Primary Outcome)	Child Asthma Control Test (CACT)
	Healthcare Use	Healthcare Visits for Pediatric Asthma (Primary Outcome)	Type of Visit for Pediatric Asthma Care: ○ Emergency Department (ED) ○ Inpatient Admission (General vs. PICU stay) ○ Outpatient (Scheduled Vs. Unscheduled) ○ Non-Revisit for Outpatient Care ○ No-Show for Outpatient Care ○ Urgent Care
